# Analysis of the Structural Aspects of Tannin-Based Adhesives by 2D-NMR

**DOI:** 10.3390/ma14195479

**Published:** 2021-09-22

**Authors:** Sachikazu Omura, Yoshinori Kawazoe, Daisuke Uemura

**Affiliations:** 1 Institute of Natural Drug-Leads, Kanagawa University, Hiratsuka 259-1293, Kanagawa, Japan; uemurad@kanagawa-u.ac.jp; 2 Center for Education and Research in Agricultural Innovation, Faculty of Agriculture, Saga University, Karatsu 847-0021, Saga, Japan; 3 Department of Chemistry, Kanagawa University, Hiratsuka 259-1293, Kanagawa, Japan

**Keywords:** adhesive, tannin, gelatin, poly-l-lysine, structure determination, 2D-NMR

## Abstract

We developed non-toxic, harmless adhesives composed of all-natural and renewable resources, of which one was composed of tannin and gelatin, which unfortunately was lacking water resistance, and the other of tannin and ε-poly-l-lysine. In this study, we analyzed the chemical structures of these adhesives by two-dimensional nuclear magnetic resonance (2D-NMR) to explain the difference in water-resistance of the two glues. The results showed that only one proton was left in the benzene ring of tannin after mixing. This suggests that the amino group of the protein was directly attached to the benzene ring by a Michael addition-type reaction, and not to the hydroxyl group. In addition, the heteronuclear multiple bond correlation spectrum of the tannin-poly-l-lysine compound indicated that the hydroxyl groups of the tannin oxidized, suggesting the improvement of its water resistance.

## 1. Introduction

We recently developed a novel non-toxic harmless adhesive composed of all-natural, renewable materials [1]. We started our study with chestnut-derived tannin, Tannin C, and animal-derived gelatin. Although the adhesive exhibited a strong gluing ability, it did not show sufficient water resistance. Then, we substituted the gelatin with a bacteria-derived ε-poly-l-lysine (EPL). The EPL-Tannin C adhesive showed such tight bonding ability and high water resistance that it was able to satisfy the European Norm EN 314 class 1. To explain the difference in resistance to water, the chemical structure of these two adhesives required clarification.

However, it is generally accepted that polymers of proteins and tannins are too complex and complicated in a mixture to determine their structure [2]. The difficulty results from the extreme heterogeneity of tannins, which is due to several kinds of esters, with different compositions of polyphenols and sugars. To overcome this problem, researchers have employed various analytical methods including mass spectrometer [3,4,5,6], SDS-PAGE [3], attenuated total reflection Fourier transform infrared (ATR-FTIR) [5,7], differential scanning calorimeter (DIC) [8,9,10,11], and the mathematical way [12]. In spite of these efforts, the exact reaction process during hardening is not yet clear, even if potential structures were deduced [13,14,15]. One of the best ways to establish their structures is X-ray crystallography or nuclear magnetic resonance (NMR) spectroscopic analysis. It is difficult, however, to attempt X-ray crystallography for the determination of adhesive structures because of their heterogeneity as stated above. In the case of NMR, almost all studies ever reported were used only for ^13^C-NMR [6,10,16,17], since it is believed that the signals are so complicated that researchers could hardly interpret the ^1^H-NMR.

**Scheme 1:**
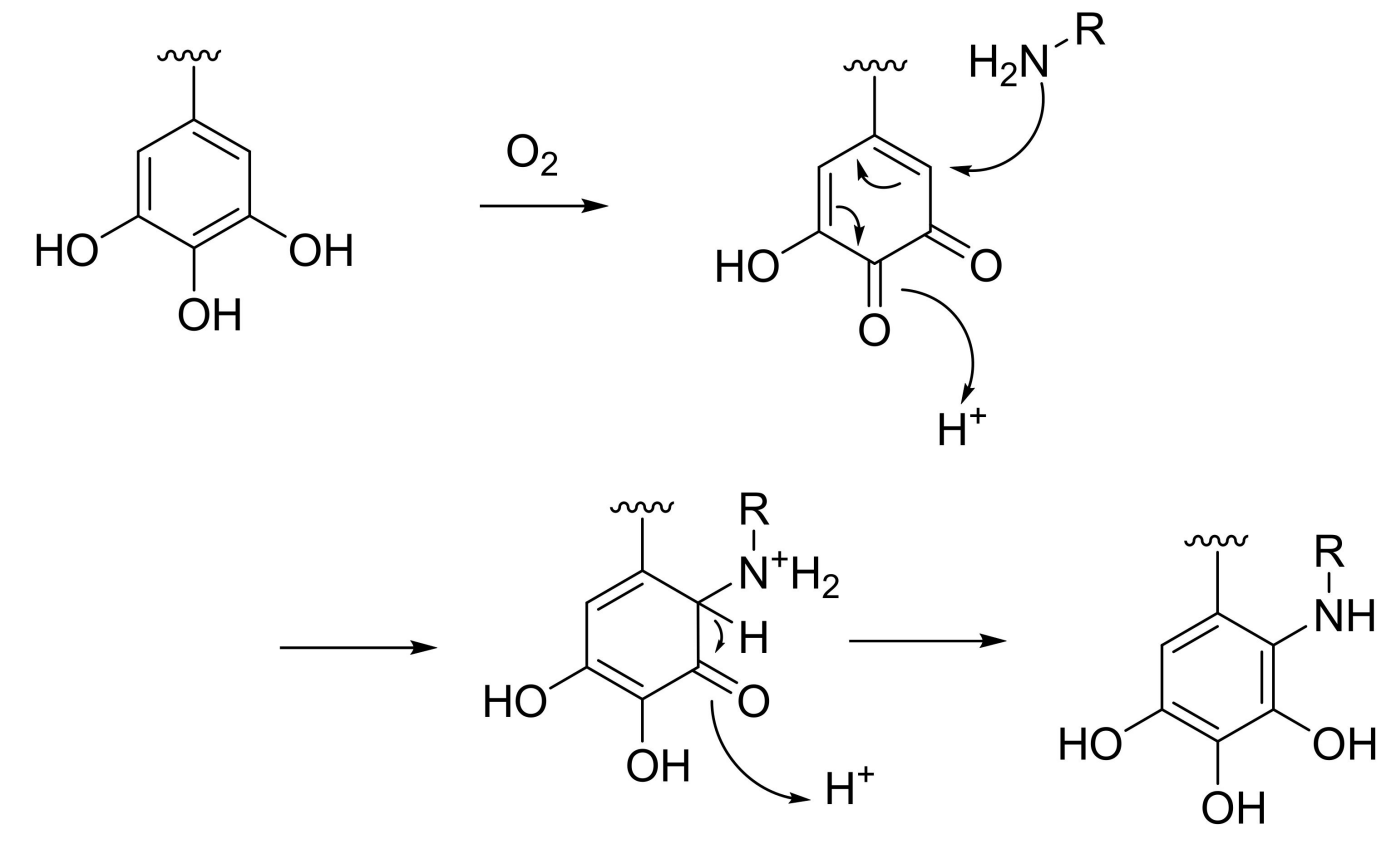
A plausible reaction process of Tannin C and the amino group of the protein/EPL.

During our previous study of tannin-based adhesives, we noticed that the oxidation process was necessary for the adhesive to harden. The observation led us to a plausible reaction pathway for Tannin C-based adhesives. In our hypothesis, the hydroxyl groups of the polyphenols of the tannin are oxidized to an *ortho*-quinone. Then, a lone pair of electrons of the nitrogen atom in the protein attacks the carbon atom of the tannin to make a covalent bond by a Michael addition-type reaction. Finally, the quinone is reduced to a phenol again. The reaction pathway stated above is visualized in Scheme 1.

Next, we wanted to verify the validity of this reaction. Therefore, it was necessary to determine the final structure. However, as stated above, there are no suitable analytical methods for this aim. This led us to attempt a totally different method, two-dimensional nuclear magnetic resonance (2D-NMR). It was impossible to assign all registered signals, but the ones concerning the benzene ring should have been clearly interpretable. Furthermore, 2D-NMR analysis enabled us to distinguish between ketone carbons and carbons that were engaged to protons or hydroxyl groups. Thus, we performed 2D-NMR analysis.

## 2. Materials and Methods

### 2.1. Reagents and Chemicals

Chestnut-derived tannin, Tannin C, was purchased from Silva Chimica (San Michele Mondovi, CN, Italy). Gelatin, tannic acid, and *ε*-poly-l-lysine were purchased from Nacalai Tesque (Kyoto, Japan). Deuterium oxide 99.9% (D_2_O; Millipore, Burlington, MA, USA) was used for the NMR.

### 2.2. Apparatus and Procedure for Recording NMR Spectra

NMR spectra were recorded by a JEOL ECA-500. The measurements used for the NMR were 10 w% solution of Tannin C, 10 w% solution of gallic acid, 10 w% solution of tannic acid, 5 w% solution of gelatin, and 5 w% solution of EPL at pH10. When recording the NMR spectrum of the gallic acid-EPL, tannic acid-EPL, Tannin C-gelatin, and Tannin C-EPL complex, a 1:1 mixture of 20 w% gallic acid, tannic acid or Tannin C solutions, and 10 w% of gelatin or EPL solutions was used.

## 3. Results

### 3.1. 2D-NMR Analysis of Simplified Model Compounds

We have developed novel adhesives composed of all-natural materials, gelatin-Tannin C and EPL-Tannin C adhesives, and we found that the latter showed strong water resistance, but the former did not. We tried to search for the key structural feature which made the difference between these two adhesives, by means of an NMR study.

**Figure 1:**
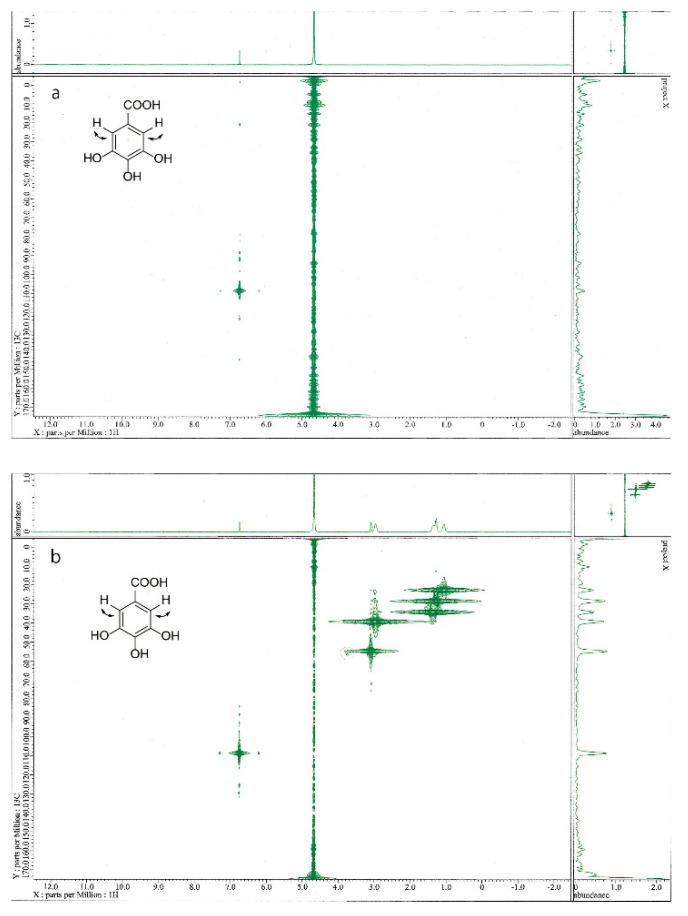
HMQC spectra of gallic acid (**a**) and gallic acid-EPL complex (**b**).

**Figure 2:**
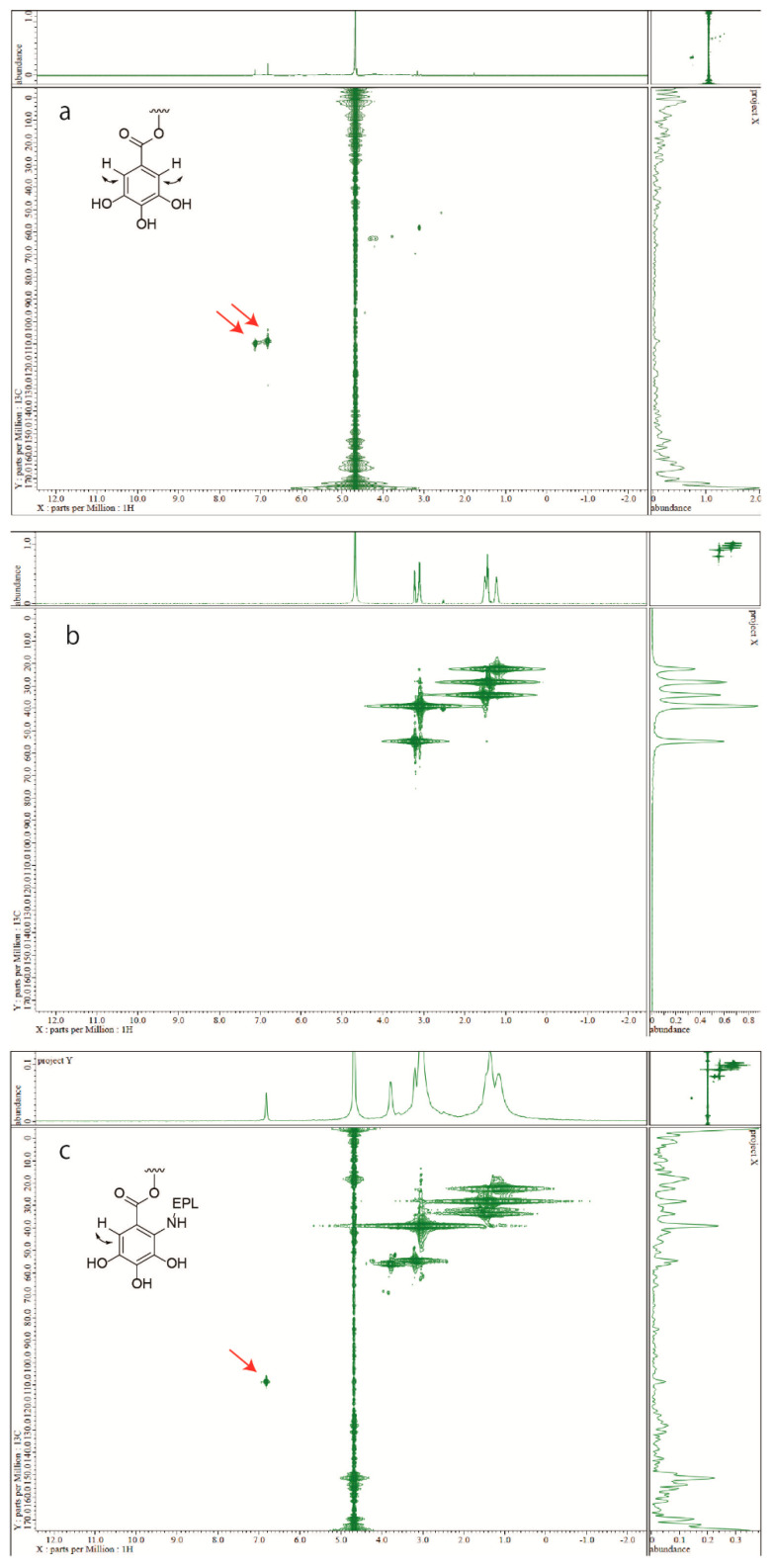
HMQC spectra of tannic acid (**a**), EPL (**b**), and their mixture (**c**).

However, it seemed difficult to interpret the NMR data, as Tannin C is a highly heterogeneous mixture of esters, gallic, and ellagic acids with sugars [2,18,19]. Judging from the hypotheses [14] and our previous study, it was obvious that the terminal polyphenol moiety reacts with the amino group of the protein or EPL. Therefore, we first selected the gallic acid as a simplified tannin model. We recorded heteronuclear multiple quantum coherence (HMQC) spectra of the gallic acid, the EPL, and their mixture in D_2_O. As expected, the gallic acid gave a single signal, which indicated the correlation of two benzene carbons and two protons (Figure 1a). Furthermore, typical signals that indicated repeating structures were observed in the case of EPL (Figure 2b). When we obtained an HMQC spectrum of their mixture, however, a remarkable change in the benzene ring could hardly be seen (Figure 1b). This was probably due to the free carboxyl group in the gallic acid, which might have reacted with the amino group in EPL. The results suggest that the gallic acid on its own was not suitable for being used in a water-resistant adhesive.

As mentioned above, the difficulty lied in the complexity of the Tannin C. When we used a homogenous material, which was an ingredient of Tannin C, we expected the analysis to become easier.

Following this idea, and based on the paper of Pizzi [13] naming all the substances contained in Tannin C, we decided on tannic acid for its commercial availability. We measured the HMQC spectra of tannic acid, EPL, and their mixture. There were two signals (6.8 ppm/108 ppm and 7.2 ppm/110 ppm) that indicated the correlation of the benzene ring carbon and protons in the tannic acid (Figure 2a, indicated by red arrows). In contrast, there was only one signal in the HMQC spectrum of the mixture (Figure 2c, indicated by a red arrow), indicating that one of the two protons of the terminal gallic acids benzene ring in the tannic acid was lost during the reaction. This could have been due to the reaction of the amino group of EPL to the carbon atom of the benzene ring by Michael addition.

### 3.2. 2D-NMR, HMQC, Analysis of Gelatin-Tannin C and EPL-Tannin C Complex

**Figure 3:**
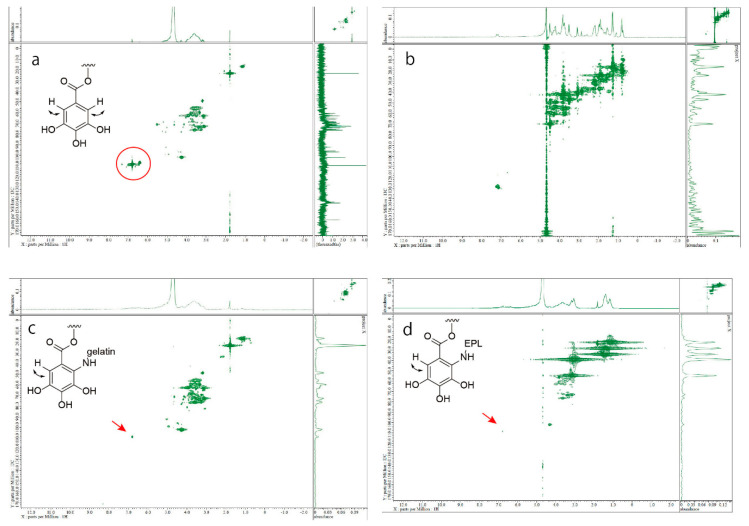
HMQC spectra of Tannin C (**a**), gelatin (**b**), gelatin-Tannin C (**c**), and EPL–Tannin C (**d**).

The above results encouraged us to analyze the 2D-NMR of the EPL-Tannin C and gelatin-Tannin C complex. Two clusters of signals were observed in the HMQC spectrum of Tannin C. One was located at 3.0~4.0 ppm/50–70 ppm and the other at 6.0~7.0 ppm/100~110 ppm. They indicated correlations between protons and carbons in the glucose- and benzene ring, respectively. The signals corresponding to the benzene ring were mainly due to the different numbers and locations of the hydroxyl groups on the components (Figure 3a, indicated by red circle). In the HMQC spectrum of gelatin, we observed a big cluster at 1.0~5.0 ppm/0~70 ppm and a small one at 7.0 ppm/130 ppm. The former seemed to correspond to the correlation of the main chain or non-aromatic side chain and the latter to the aromatic side chain (Figure 3b).

Next, we recorded an HMQC spectrum after mixing Tannin C with gelatin or EPL. This showed, as expected, that the carbohydrates of the Tannin C stayed intact, while there were changes in the benzene ring. Of the several signals recorded at 6.0~7.0 ppm/100~110 ppm in the HMQC spectrum of Tannin C (Figure 3a, indicated by a red circle), most disappeared and only one signal was left in the spectrum of the mixture of Tannin C with gelatin or EPL (Figure 3c,d, respectively, indicated by red arrows). These results clearly indicated that there was only one proton on the benzene ring, suggesting that the amino group of EPL or the protein directly attached to the benzene ring, but not to the hydroxyl group of the gallic acid.

### 3.3. 2D-NMR, HMBC, Analysis of EPL-Tannic Acid Complex

**Figure 4:**
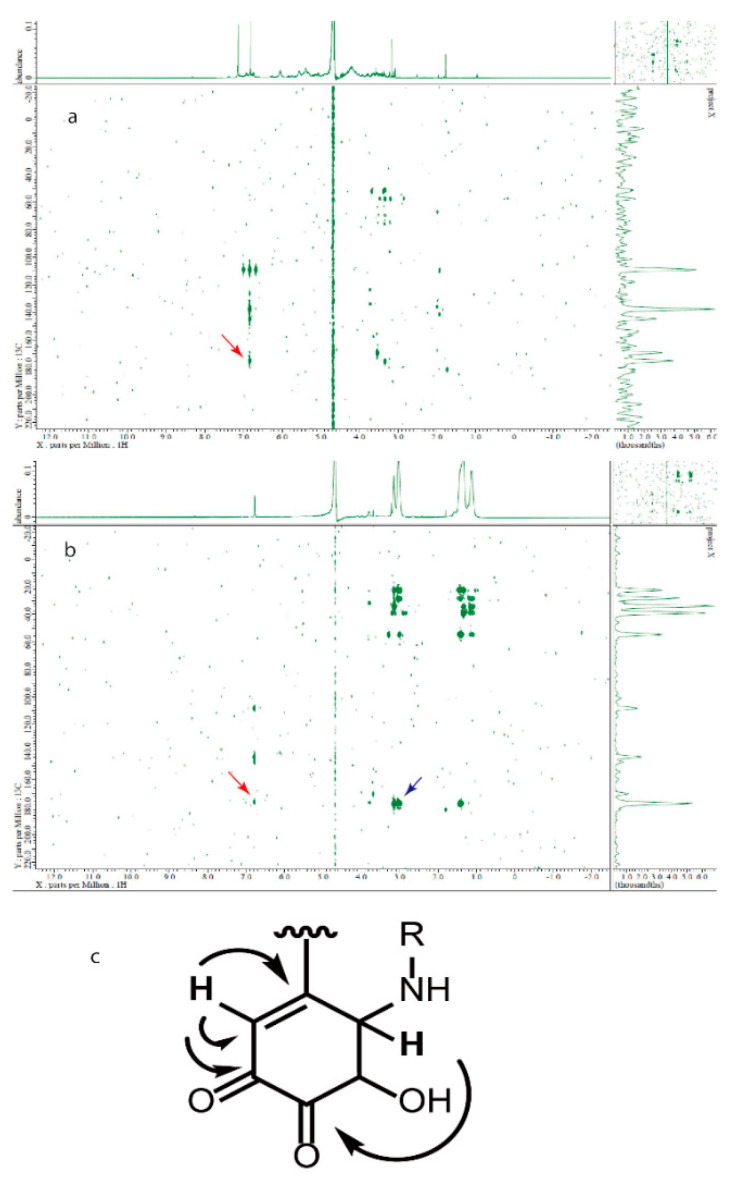
HMBC spectrum of tannic acid (**a**) and EPL-tannic acid (**b**). (**c**) shows a summary of HMBC spectra of EPL–tannic acid.

Finally, we investigated if the two ketone groups in the quinone ring were reduced or not. Therefore, we recorded the heteronuclear multiple bond correlation (HMBC) spectra of tannic acid and of the mixture of tannic acid and EPL. Judging from the HMBC spectrum of tannic acid, some of the hydroxyl groups should have been oxidized, as there was a correlation between the proton on the quinone ring and the carbonyl carbons (Figure 4a, indicated by a red arrow.) A remarkable feature of the HMBC spectrum, of the tannic acid and EPL mixture, was the correlation between the proton and the carbonyl carbons (Figure 4b, indicated by a red arrow). This suggested the existence of a carbonyl carbon in the quinone ring. In addition, the increase in the number of protons which were correlated with the carbonyl carbons was observed. This indicated the correlation of protons and carbonyl carbon in the main chain of EPL. The signal at 3.00 ppm/180 ppm (Figure 4b, indicated by a blue arrow), however, was not corresponding to the EPL main chain, but more probably to the correlation between the proton on the quinone ring and the carbonyl carbon. Figure 4c summarizes these data.

## 4. Discussion

In summary, we successfully determined the partial chemical structure of a tannin-based adhesive by conducting 2D-NMR, HMQC, and HMBC tests. Although many polymer structures of tannin-based adhesives have been proposed so far [13,14,15], they all were deduced through mass spectrometry, FT-IR, or the mathematical way. As far as we know, this is the first report that shows the NMR-defined structure of a tannin-based adhesive. Our results of the 2D-NMR analyses proved the viability of our hypothesis that oxidation is important for the reaction of the Tannin C with proteins, and verified our hypothetical reaction pathway, demonstrating that the amino group of the protein/EPL did not condense to a hydroxyl group on the benzene ring but reacted directly with the benzene carbon atom of the gallic acid. Furthermore, this Michael addition-type reaction between Tannin C and gelatin strongly suggested that the production of the *ortho*-quinone was an intermediate of the gallic acid oxidation, even though we could not detect it.

**Scheme 2:**
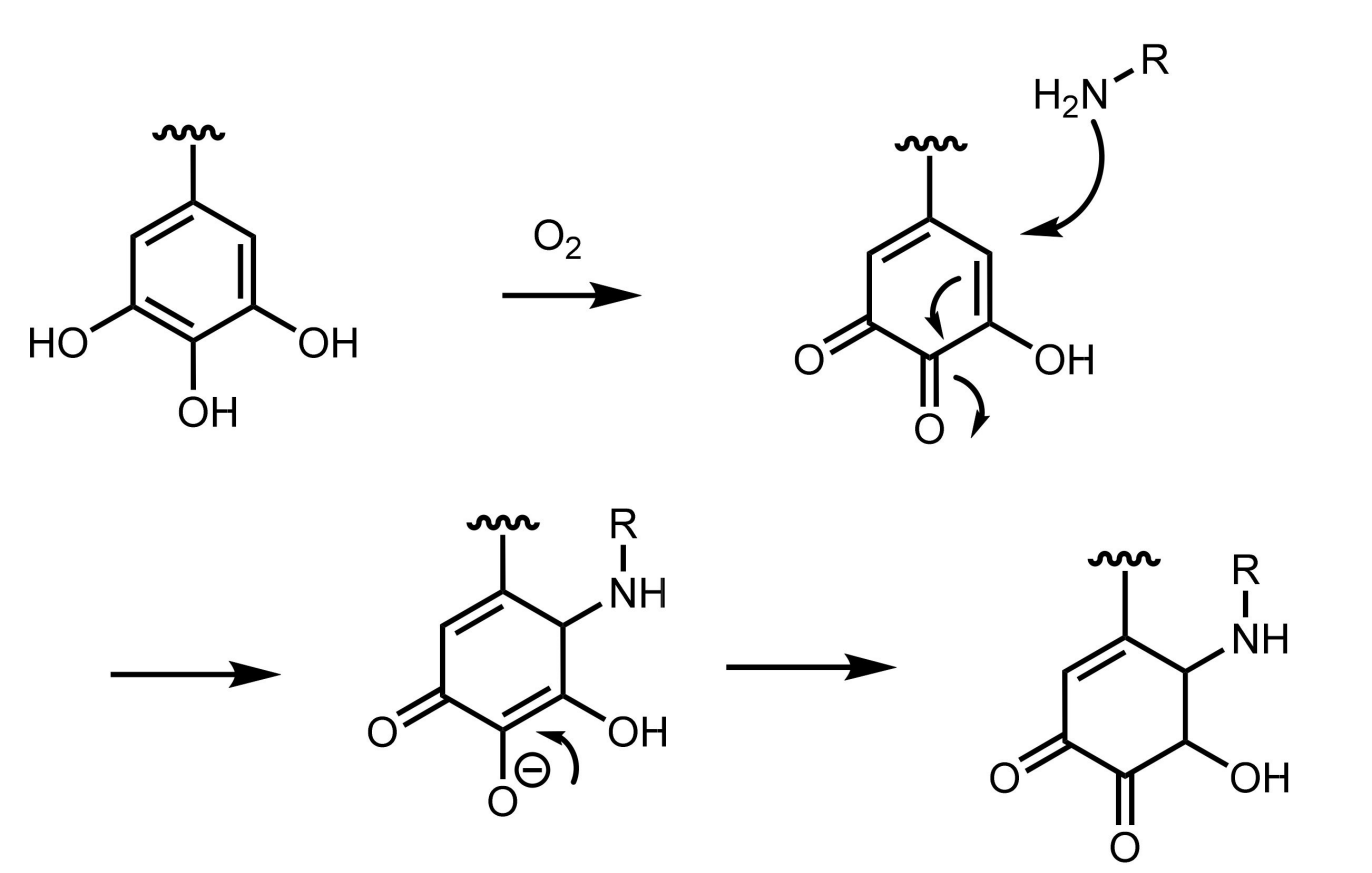
Revised version of the reaction pathway of an EPL-Tannin C complex.

Alongside others [13,14,15], we proposed the possible reaction products of tannin with an amino group of a protein. They, however, propose that the terminal gallic acid moiety is oxidized to an *ortho*-quinone, and then stays the same or reduces to re-produce polyphenol. This is based on the observation of the importance of the oxidation process or determination of a tiny part-structure of the product. Our experiments, on the other hand, clearly demonstrated that the final product was a cyclohexanedione derivative (Scheme 2).

The Tannin C-EPL adhesive showed excellent water resistance, even if one hydroxyl group and two ketone groups remained intact. Thus far, we are not able to explain this discrepancy. One possibility is a deletion of the ketone groups by Schiff base formation with the amino groups of EPL. It was not possible to record NMR spectra after the completion of the reaction, as the NMR method could not be applied once the adhesive had hardened. Therefore, all our spectra were measured in the early stage of the reaction. To clarify the hardened structure of the adhesives, we could try to conduct solid-state NMR with ^15^N-labeled EPL-Tannin C complex.

Li et al. proposed a possible reaction between condensed tannin and the polyethyleneimine (PEI) [14]. According to their hypothesis, the terminal polyphenol in tannin is oxidized, then reacts with the amino group of the PEI by a Michael addition type-reaction, and forms a Schiff base formation with ortho-quinones. Our findings experimentally substantiated their hypothesis that a Michael addition type-reaction took place in the mixture of tannin and proteins. However, their last process is different from ours: we propose that the quinones were reduced to hydroxyl groups (Scheme 1). To resolve this discrepancy, it would be necessary to determine the total structure of the polymer in detail.

It is the general belief that it is difficult to determine the structure of a heterogeneous mixture such as a tannin-gelatin adhesive. Contrary to this, we conducted 2D-NMR to analyze the structure of our Tannin C and gelatin adhesive. As far as we know, this is the first report to determine the structure directly, even if it was a partial structure, of a tannin-based adhesive by the use of 2D-NMR. Although we were not able to assign all of the signals, we successfully identified the reaction site of Tannin C with gelatin or EPL. Our achievement partly depended on our hypothesis, which made us focus on the aromatic ring of Tannin C. Thus, 2D-NMR might be an effective method to analyze the structure of a tannin-based adhesive, if you know on which functional group you need to focus. This report describes a case study with adhesive of Tannin C and gelatin, but our efforts may serve as a proof-of-principle that points out the effectiveness of 2D-NMR in determination of structure of heterogeneous mixtures.

## Data Availability

The data presented in this study are available in the Supplementary Materials.

## Supplementary Materials

The following are available online at https://www.mdpi.com/article/10.3390/ma14195479/s1, Higher resolution and large size of Figure 1, Figure 2, Figure 3 and Figure 4.
