# Supplementary material for: Analysis of the Structural Aspects of Tannin-Based Adhesives by 2D-NMR
Source: Materials (Basel). 2021 Sep 22;14(19):5479. doi: 10.3390/ma14195479 (PMC8509686; doi:10.3390/ma14195479)
Supplement: Supplementary file 1 [file materials-14-05479-s001.zip › materials-1341382-supplementary.pdf]

Supplementary Materials

Analysis of the structural aspects of tannin-based adhesives by 2D-NMR

Sachikazu Omura[1]*, Yoshinori Kawazoe[2]*, and Daisuke Uemura[1,3]

[1]Institute of Natural Drug-Leads, Kanagawa University

[2]Center for Education and Research in Agricultural Innovation, Faculty of Agriculture, Saga University

[3]Department of Chemistry, Kanagawa University

*Corresponding author: Tel.:+81-463-5911, sachi@omura-zh.ch (S.O.)

*Corresponding author: Tel.:+81-955-77-4484, ykawazoe@cc.saga-u.ac.jp (Y.K.)

Higher Resolution Images of NMR spectra in Figures 1–4

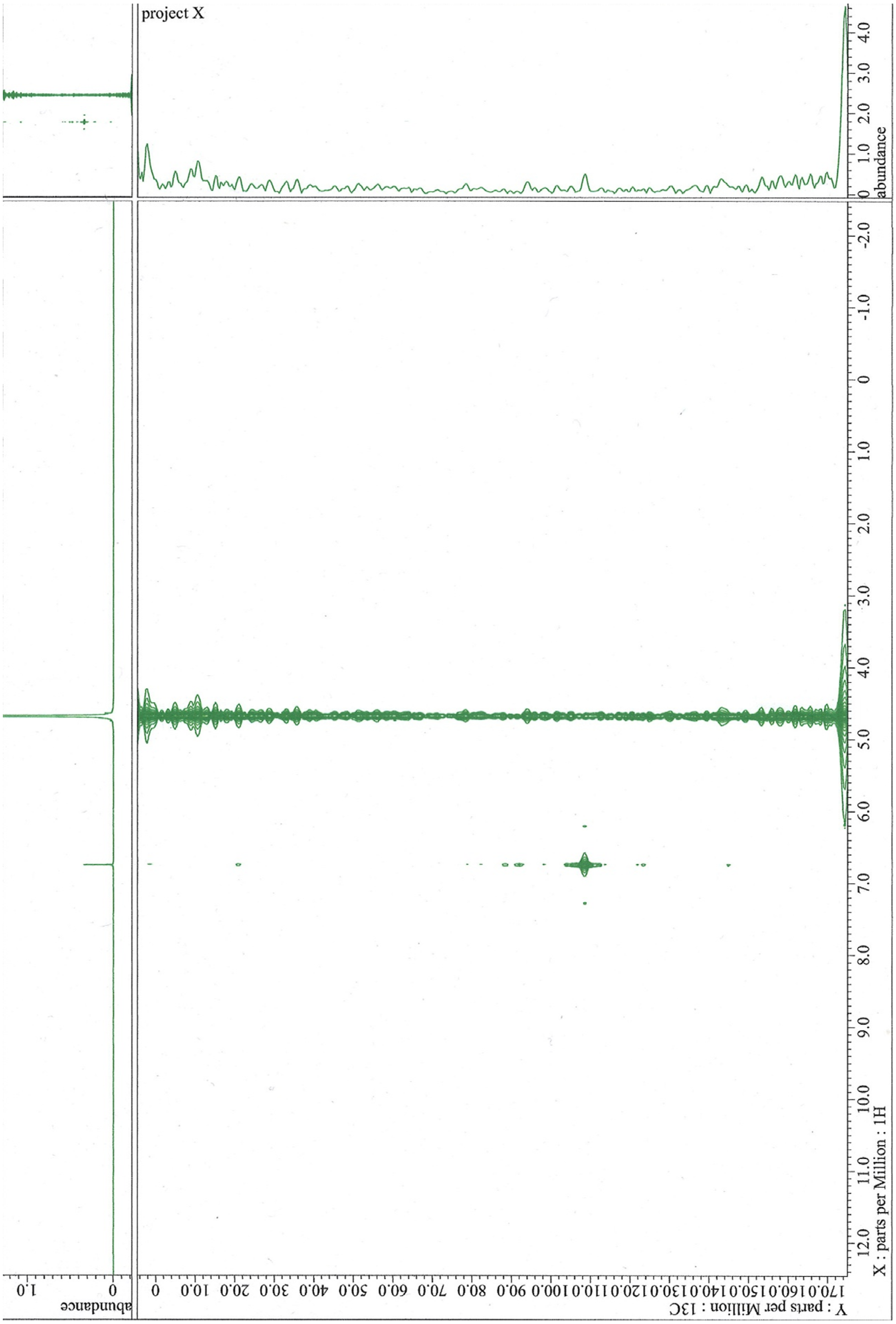
project X
abundance
X : parts per Million : 1H
Y : parts per Million : 13C
abundance

Figure1a

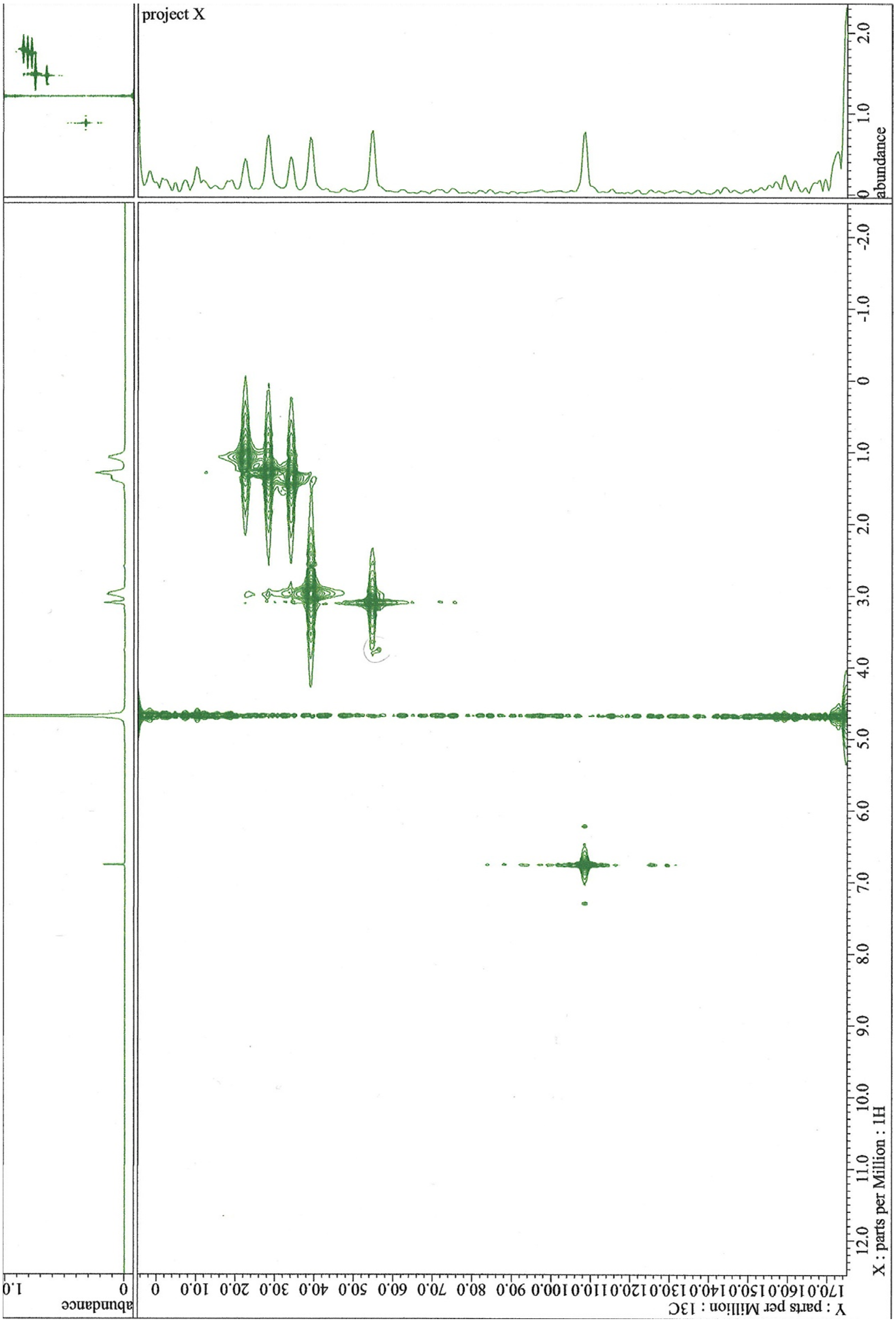
project X
abundance
X : parts per Million : 1H
Y : parts per Million : 13C
abundance

Figure 1b

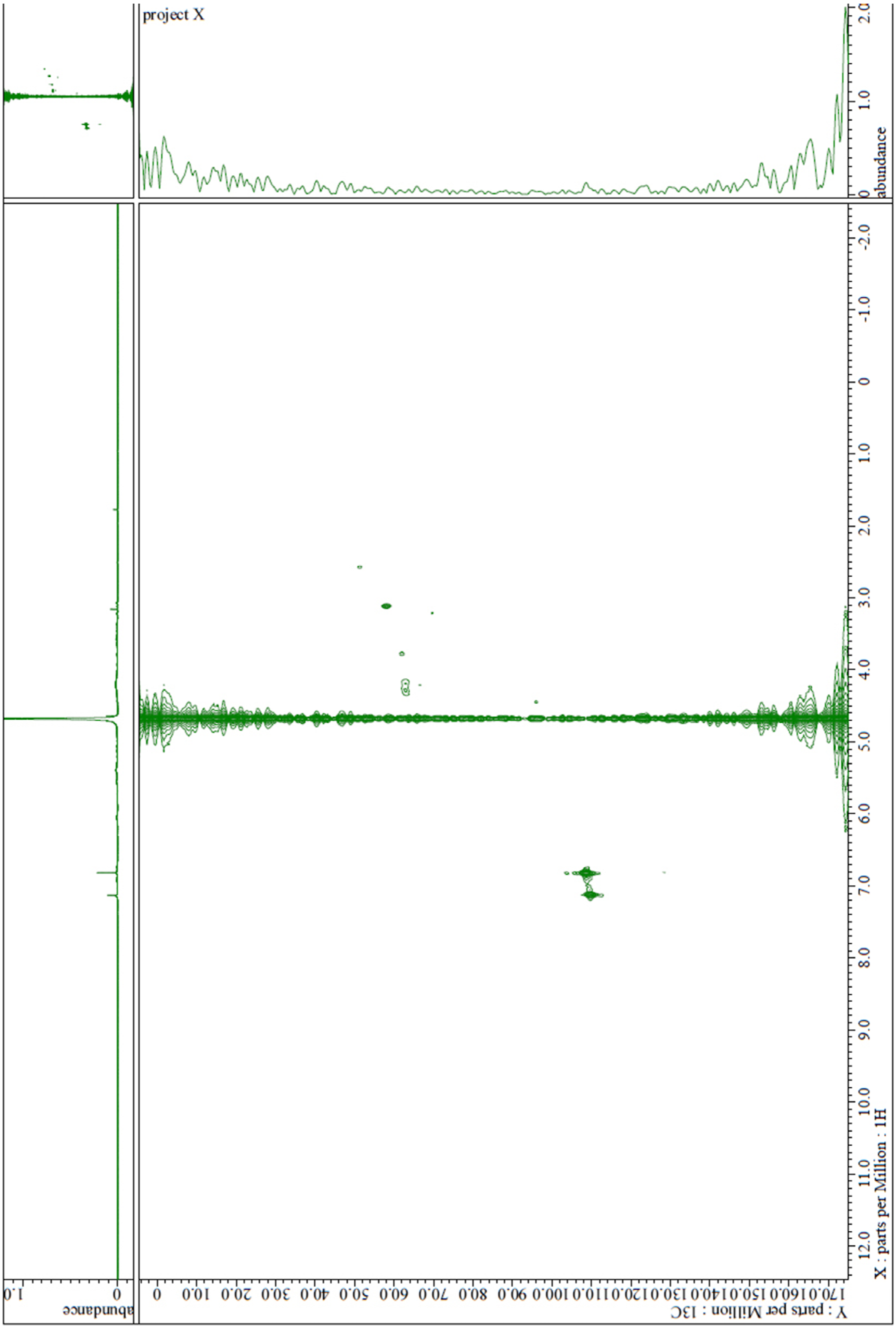
project X
abundance
X : parts per Million : 1H
Y : parts per Million : 13C
abundance

Figure 2a

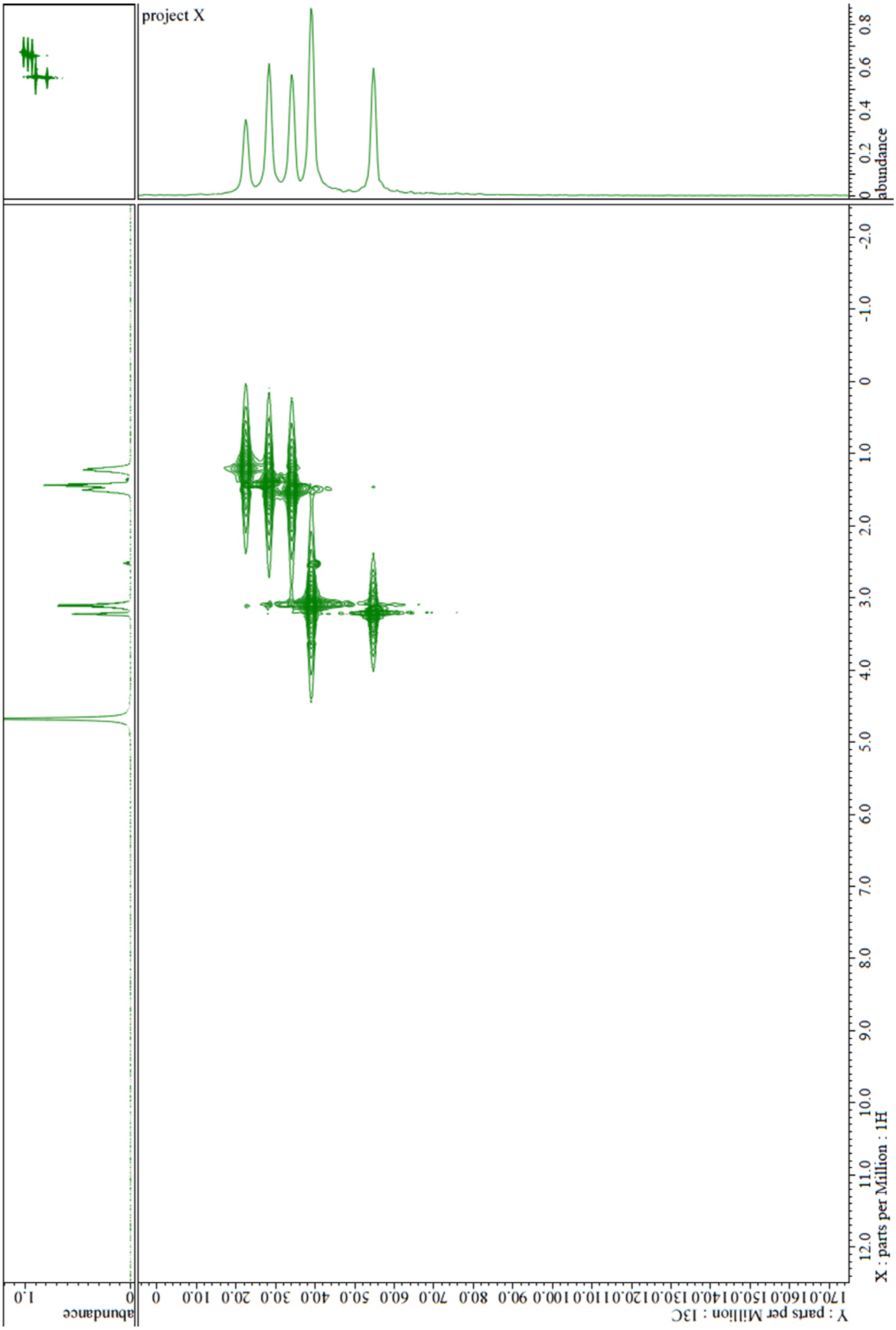
project X
abundance
X : parts per Million : 1H
Y : parts per Million : 13C
abundance

Figure 2b

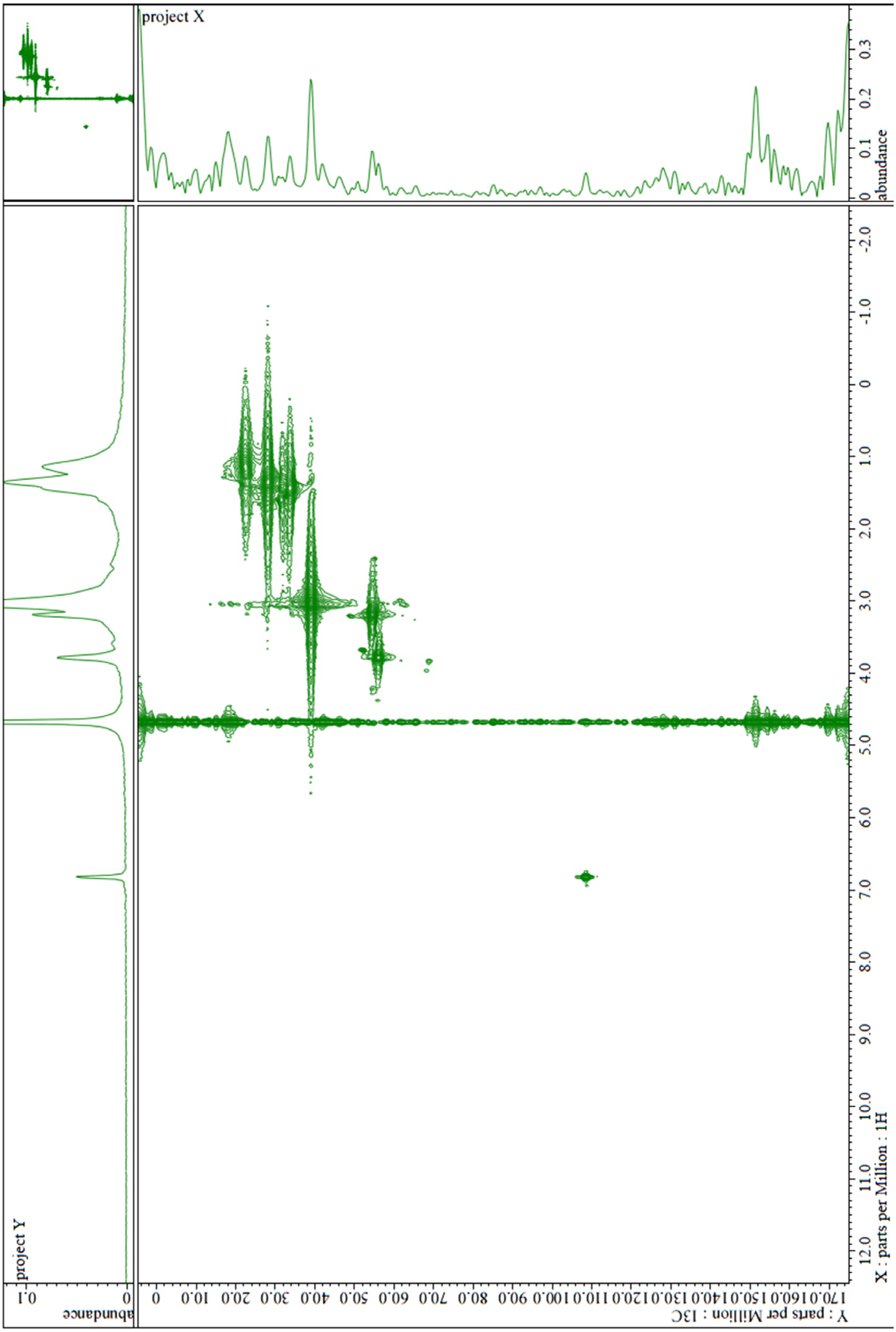
project X
project Y
abundance
abundance
X : parts per Million : 1H
Y : parts per Million : 13C

Figure 2c

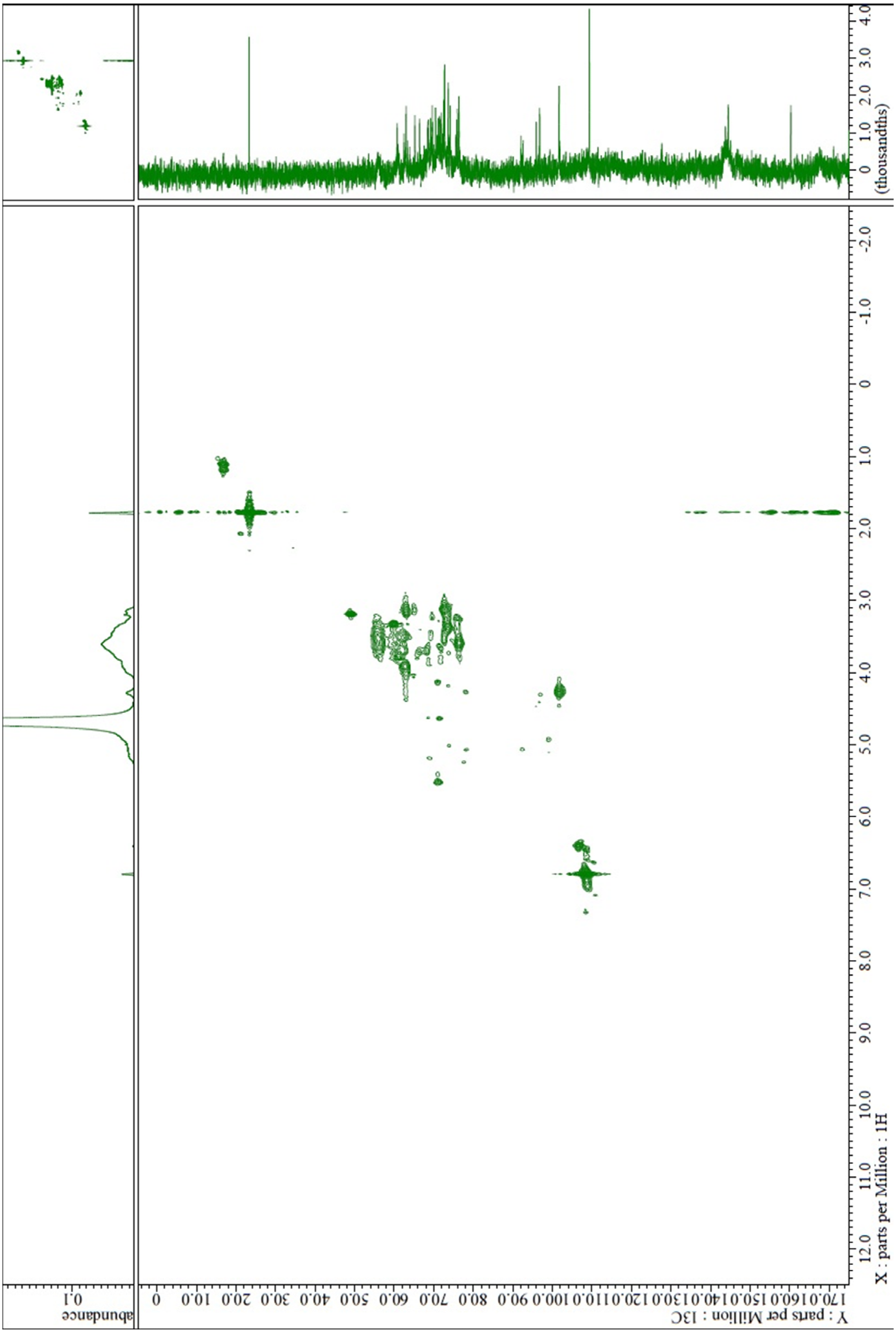
X : parts per Million : 1H
Y : parts per Million : 13C
(thousandths)
abundance

Figure 3a

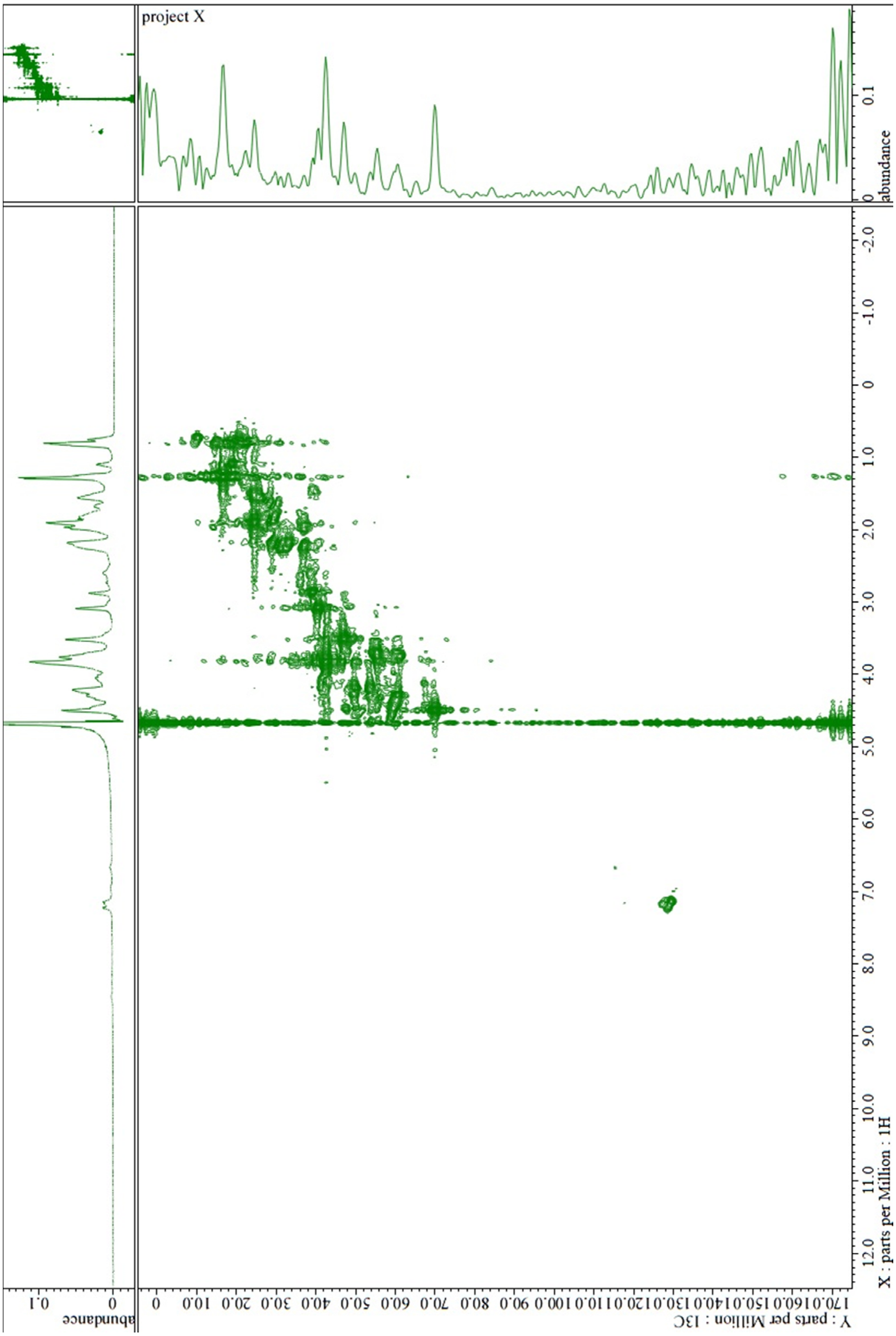
project X
abundance
X : parts per Million : 1H
Y : parts per Million : 13C

Figure 3b

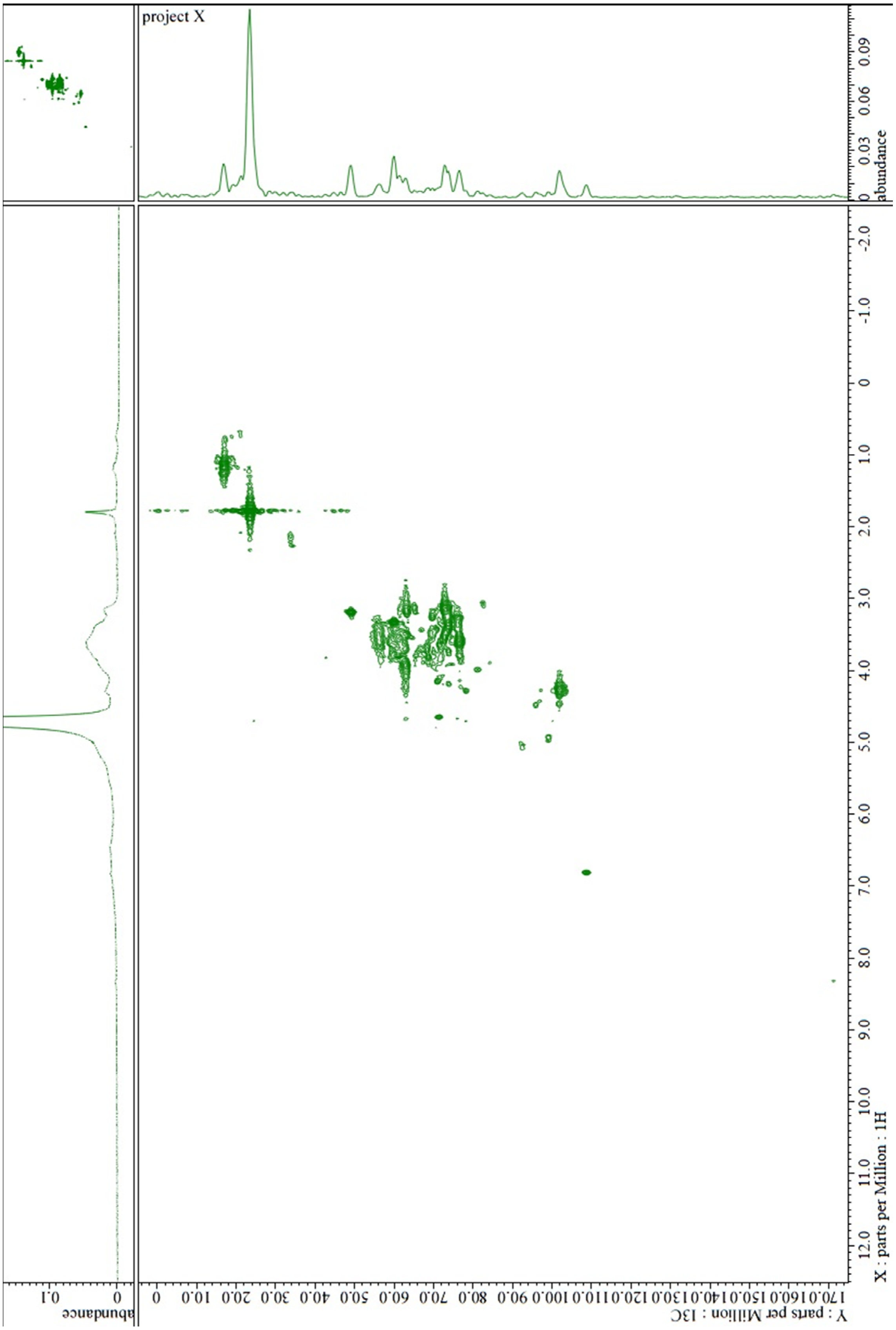
project X
abundance
X : parts per Million : 1H
Y : parts per Million : 13C

Figure 3c

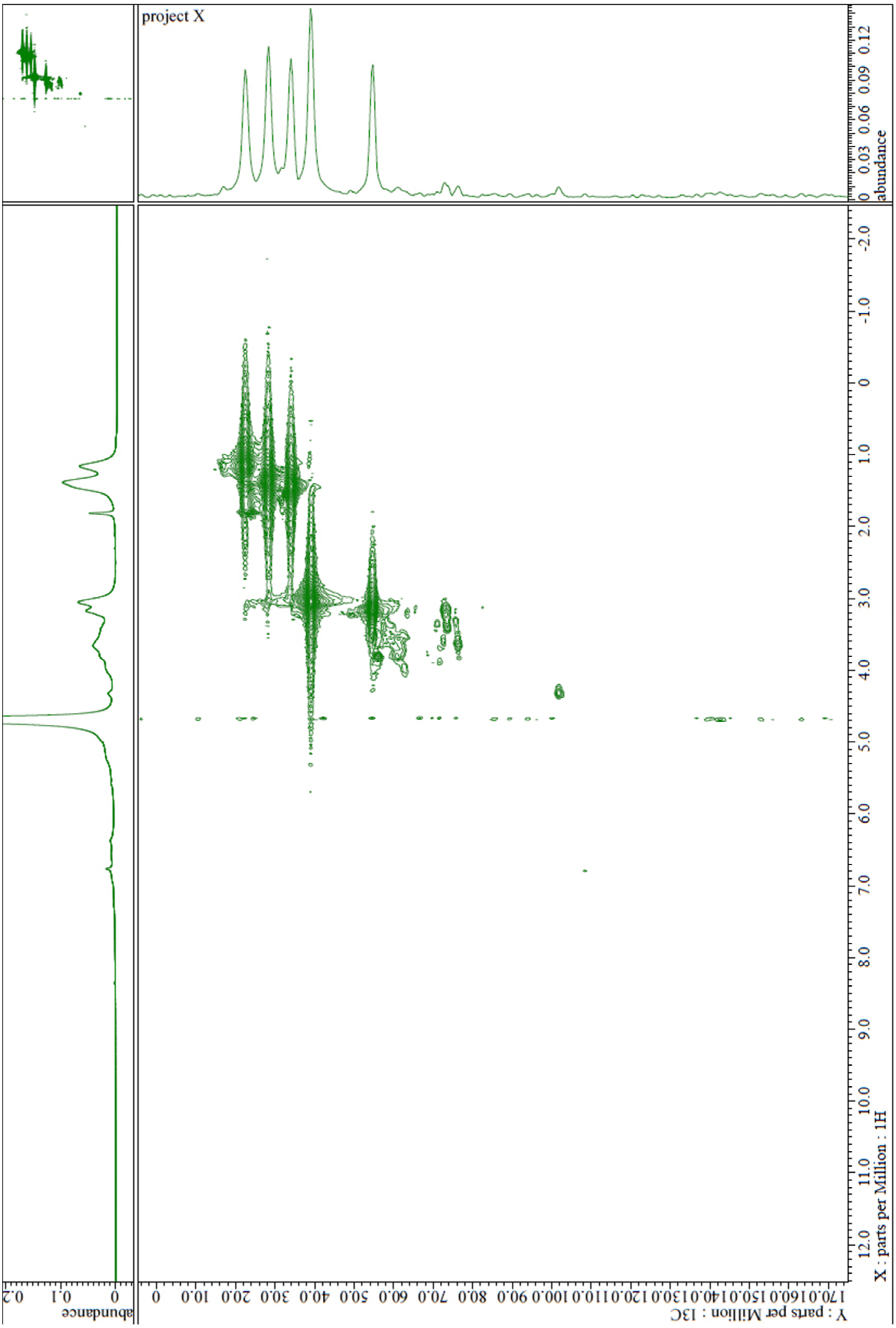

project X
abundance
X : parts per Million : 1H
Y : parts per Million : 13C
abundance

Figure 3d

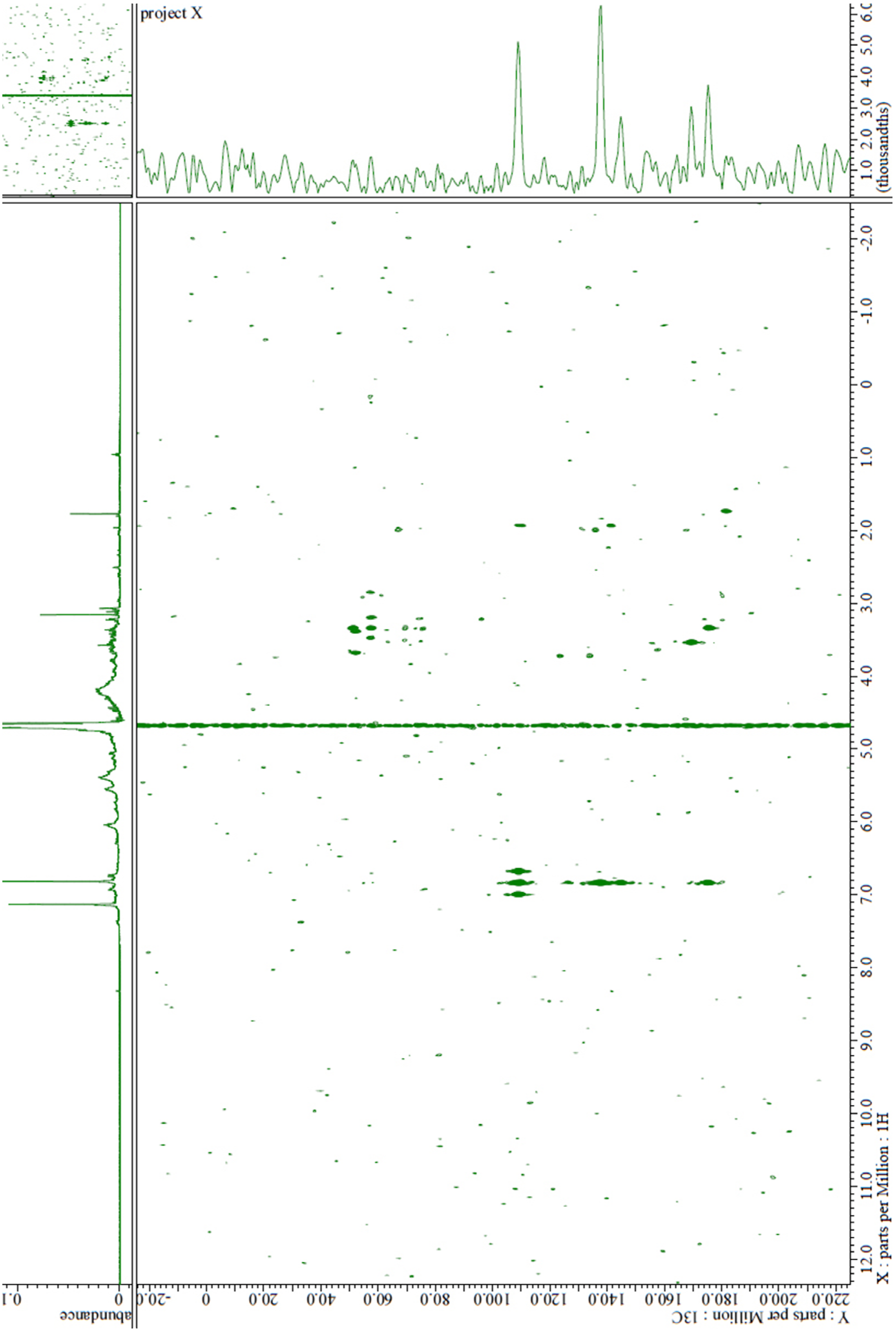
project X
(thousandths)
X : parts per Million : 1H
Y : parts per Million : 13C
abundance

Figure 4a

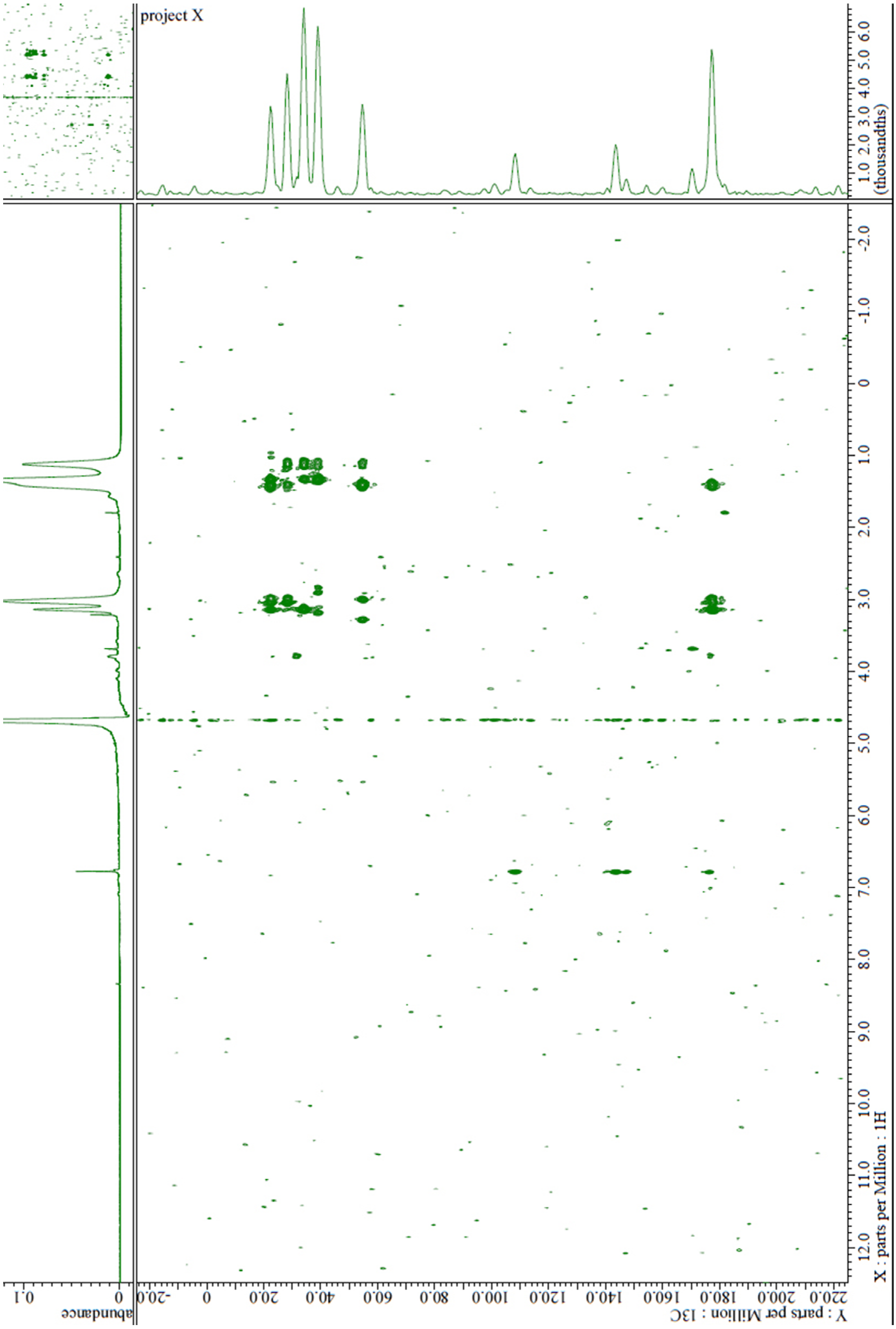
project X
X : parts per Million : 1H
Y : parts per Million : 13C
abundance
(thousandths)

Figure 4b
